# Author Correction: Mitophagy curtails cytosolic mtDNA-dependent activation of cGAS/STING inflammation during aging

**DOI:** 10.1038/s41467-024-46010-7

**Published:** 2024-02-19

**Authors:** Juan Ignacio Jiménez-Loygorri, Beatriz Villarejo-Zori, Álvaro Viedma-Poyatos, Juan Zapata-Muñoz, Rocío Benítez-Fernández, María Dolores Frutos-Lisón, Francisco A. Tomás-Barberán, Juan Carlos Espín, Estela Area-Gómez, Aurora Gomez-Duran, Patricia Boya

**Affiliations:** 1 Department of Cellular and Molecular Biology, Centro de Investigaciones Biológicas Margarita Salas, CSIC, Madrid, Spain; 2 Department of Neuroscience and Movement Science, Section of Medicine, University of Fribourg, Fribourg, Switzerland; 3 Food & Health Lab, Research Group on Quality, Safety, and Bioactivity of Plant Foods, CEBAS-CSIC, Murcia, Spain; 4 Department of Biomedicine, Centro de Investigaciones Biológicas Margarita Salas, CSIC, Madrid, Spain; 5 MitoPhenomics Lab, Centro Singular de Investigación en Medicina Molecular y Enfermedades Crónicas, Universidade de Santiago de Compostela, Santiago de Compostela, Spain

**Keywords:** Autophagy, Cell death, Mitochondria, Inflammation

Correction to: *Nature Communications* 10.1038/s41467-024-45044-1, published online 27 January 2024

The original version of this article contained an error in Fig. 7a. The representative image for ABT-737+QVD+UA (Scr-siRNA) was inadvertently duplicated from the image for Control. The image for ABT-737+QVD+UA (Scr-siRNA) has been replaced with a correct image. This has now been corrected in the HTML and PDF version of this Article.

