# Supplementary material for: Mitophagy curtails cytosolic mtDNA-dependent activation of cGAS/STING inflammation during aging
Source: Nat Commun. 2024 Jan 27;15:830. doi: 10.1038/s41467-024-45044-1 (PMC10821893; doi:10.1038/s41467-024-45044-1)
Supplement: Supplementary file 1 — Supplementary Information [file 41467_2024_45044_MOESM1_ESM.pdf]

Supplementary Information for:

Mitophagy curtails cytosolic mtDNA-dependent activation of cGAS/STING inflammation during aging

Juan Ignacio Jiménez-Loygorri[1], Beatriz Villarejo-Zori[1], Álvaro Viedma-Poyatos[1], Juan Zapata-Muñoz[1], Rocío Benítez-Fernández[1,2], María Dolores Frutos-Lisón[3], Francisco A. Tomás-Barberán[3], Juan Carlos Espín[3], Estela Area-Gómez[1], Aurora Gómez-Durán[4,5], Patricia Boya[1,2*]

Corresponding author: patricia.boya@unifr.ch

The PDF file includes:

Supplementary Figures 1 to 10

Supplementary Table 1

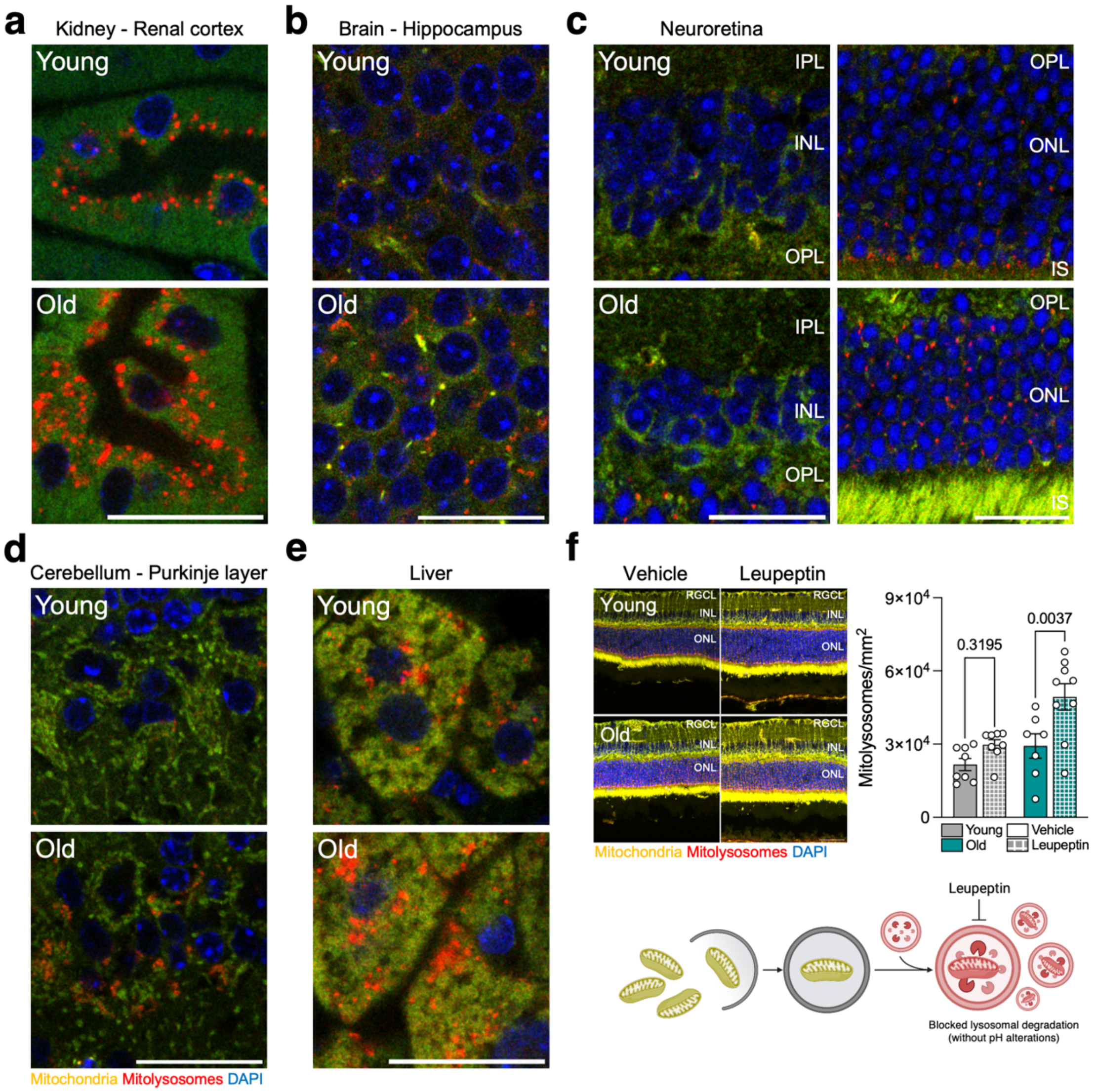

Supplementary Fig. 1. Mitophagy during physiological aging. High magnification images from Fig.1 of young (6–8 months) and old (24–26 months) *mito*-QC reporter mice bred on a C57Bl6/J background. **a** Kidney, renal cortex. **b** Brain, hippocampus. **c** Neuroretina, inner nuclear layer (INL, left) and outer nuclear layer (ONL, right). **d** Cerebellum, Purkinje layer. **e** Liver. **f** Young (8 months) and old (26 months) *mito*-QC mice were administered a single intraperitoneal injection of protease inhibitor Leupeptin (40 mg/kg) or vehicle (saline). Representative images and quantification of mitolysosome number are shown (n = 7-9 mice).

Scale bars, 25 μm (**a**-**e**) and 50 μm (**f**). All data are expressed as the mean ± s.e.m. Dots represent independent experiments. *P* values were calculated using 2-way ANOVA with Šídák's *post-hoc* test (**f**). Created with BioRender.com Source data are provided as a Source Data file.

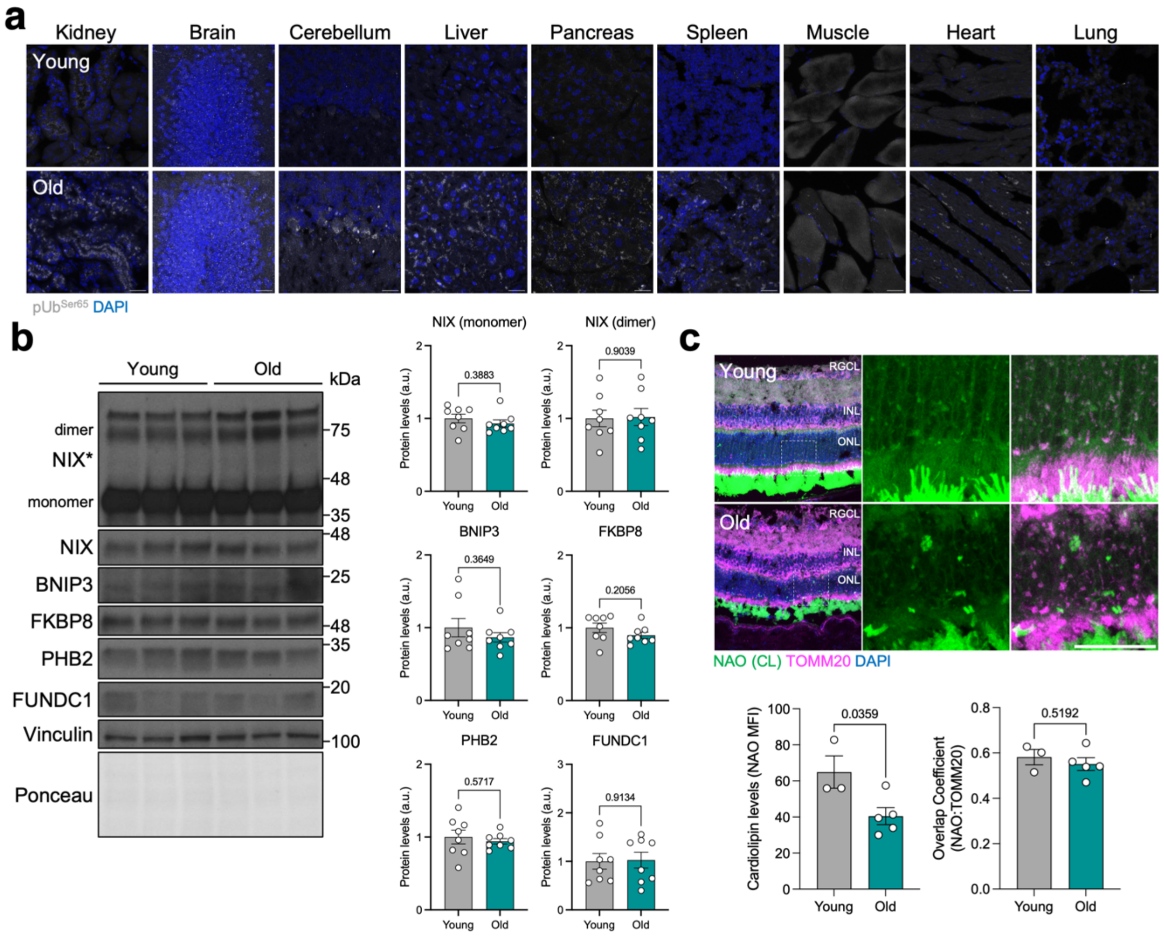

Supplementary Fig. 2. Mitophagy pathways during physiological aging. a Representative images showing phospho-Ubiquitin^{Ser65} (gray) immunostaining of cryosections of the kidney, brain, cerebellum, liver, pancreas, spleen, muscle, heart, and lung from young and old mice, as shown in Fig. 1d. **b** Western blot analysis of mitophagy receptors (BNIP3L/NIX, BNIP3, FKBP8, PHB2, FUNDC1) in the retinas of young and old mice (n = 8 mice). Vinculin was used as a loading control. Asterisk indicates higher exposure for chemiluminescence detection. **c** Immunostaining for mitochondria (TOMM20, magenta) and cardiolipin (CL) labelling (Nonyl acridine orange; NAO, green): representative images and corresponding quantification of CL levels and TOMM20:CL colocalization (n = 3-5 mice). Scale bars, 25 µm (**a**, **c**). All data are

expressed as the mean ± s.e.m. Dots represent individual mice. *P* values were calculated using a two-tailed Student's *t*-test. Source data are provided as a Source Data file.

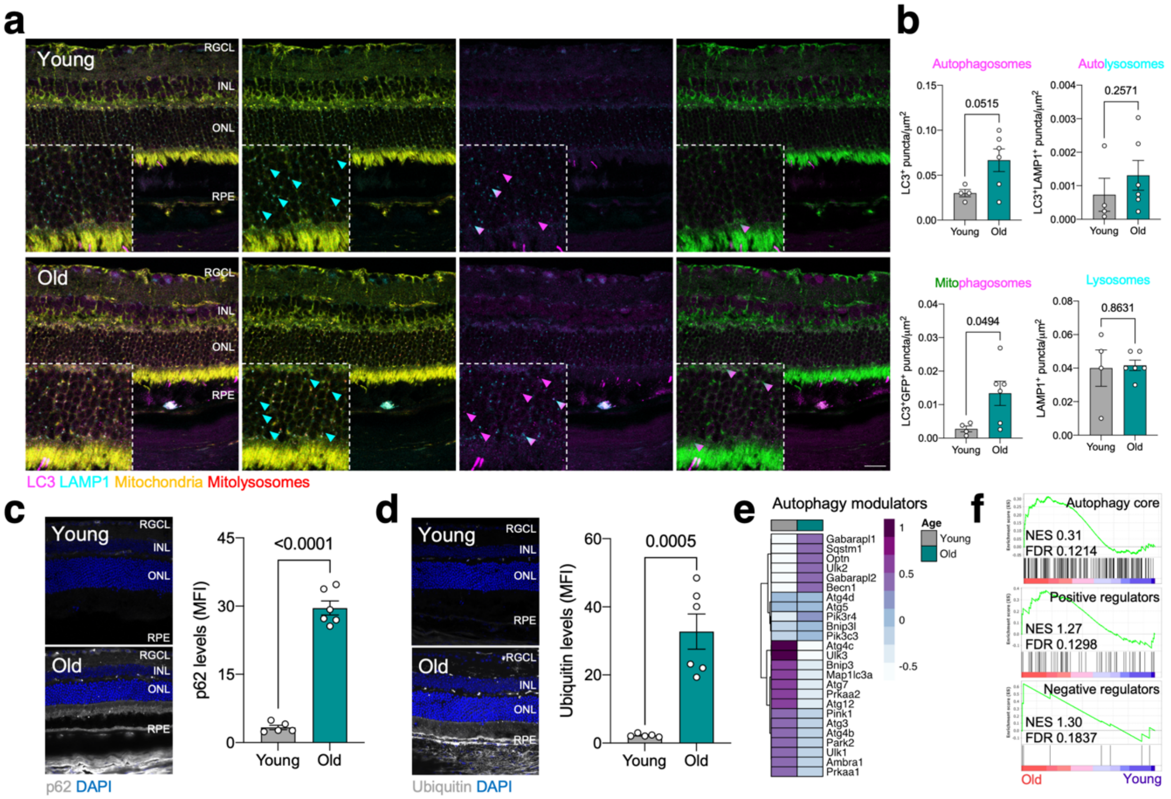

Supplementary Fig. 3. Macroautophagy during physiological aging. a Representative images showing immunostaining for LC3 (magenta, autophagic vesicles) and LAMP1 (cyan, lysosomes) in whole eye cryosections from young and old *mito*-QC mice. **b** Quantification of the number of autophagosomes (LC3^{+}), autolysosomes (LC3^{+}LAMP1^{+}), mitophagosomes (LC3^{+}GFP^{+}), and lysosomes (LAMP1^{+}) in a (*n* = 7-9 mice). **c** Immunostaining for SQSTM1/p62 (grey): representative images and corresponding quantification of whole eye cryosections from young and old mice (*n* = 5-6 mice). **d** Immunostaining for Ubiquitin (grey): representative images and corresponding quantification of whole eye cryosections from young and old mice (*n* = 5-6 mice). **e** Heatmap depicting the expression levels of autophagy and mitophagy regulators in the retina of individual young and old mice. **f** Enrichment analysis of core machinery, positive and negative regulators of autophagy. Scale bars, 25 μm (**a**, **c**, **d**). All data are expressed as the mean ± s.e.m.

Dots represent individual mice. *P* values were calculated using a two-tailed Student's *t*-test (**b** [autophagosomes, mitophagosomes, lysosomes], **c**, **d**) or two-tailed Mann-Whitney *U*-test (**b** [autolysosomes]). Source data are provided as a Source Data file.

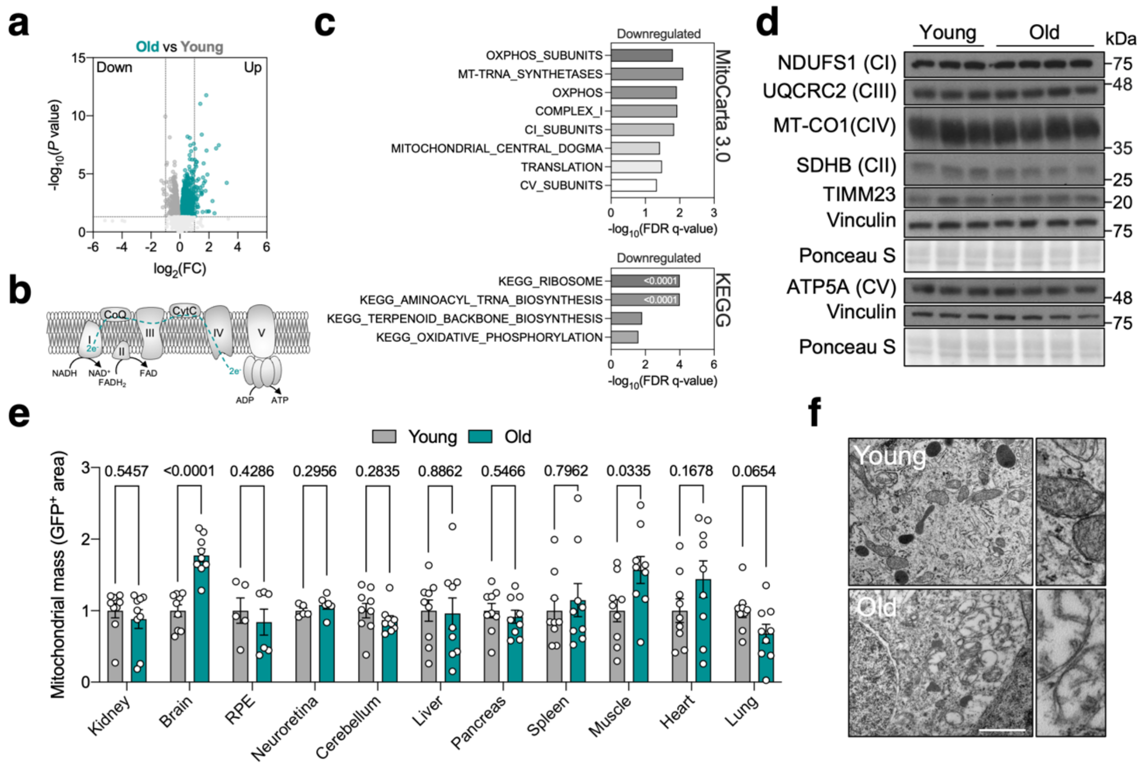

Supplementary Fig. 4. No gross alterations in mitochondria levels are observed in old versus young mice. a Volcano plot depicting differentially-expressed genes (DEGs) in young and old mouse retinas. **b** Diagram showing the main components and substrates of the mitochondrial electron transport chain (ETC). **c** Enrichment analysis showing MitoCarta 3.0 (top) and KEGG (bottom) pathways that are downregulated in old versus young retinas. **d** Western blot analysis of mitochondrial ETC components (CI-NDUFS1, CII-SDHB, CIII-UQCRC2, CIV-MT-CO1, CV-ATP5A) and total mitochondrial mass (TIMM23) in the retinas of young and old mice. Vinculin was used as a loading control. **e** Mitochondrial mass analysis of Fig. 1B, identified as the GFP+ area corresponding to cytoplasmic mitochondria (*n* = 5-9 mice). **f** Representative electron microscopy images of the retina of young and old mice. Insets show

higher magnification images of mitochondria. Scale bar, 2 μm (**f**). All data are expressed as the mean ± s.e.m. Dots represent individual mice. *P* values were calculated using the two-tailed Student's *t*-test (**e**, brain, neuroretina, cerebellum, liver, pancreas, muscle, heart, lung) or two-tailed Mann-Whitney *U*-test (**e**, kidney, RPE, spleen). Source data are provided as a Source Data file.

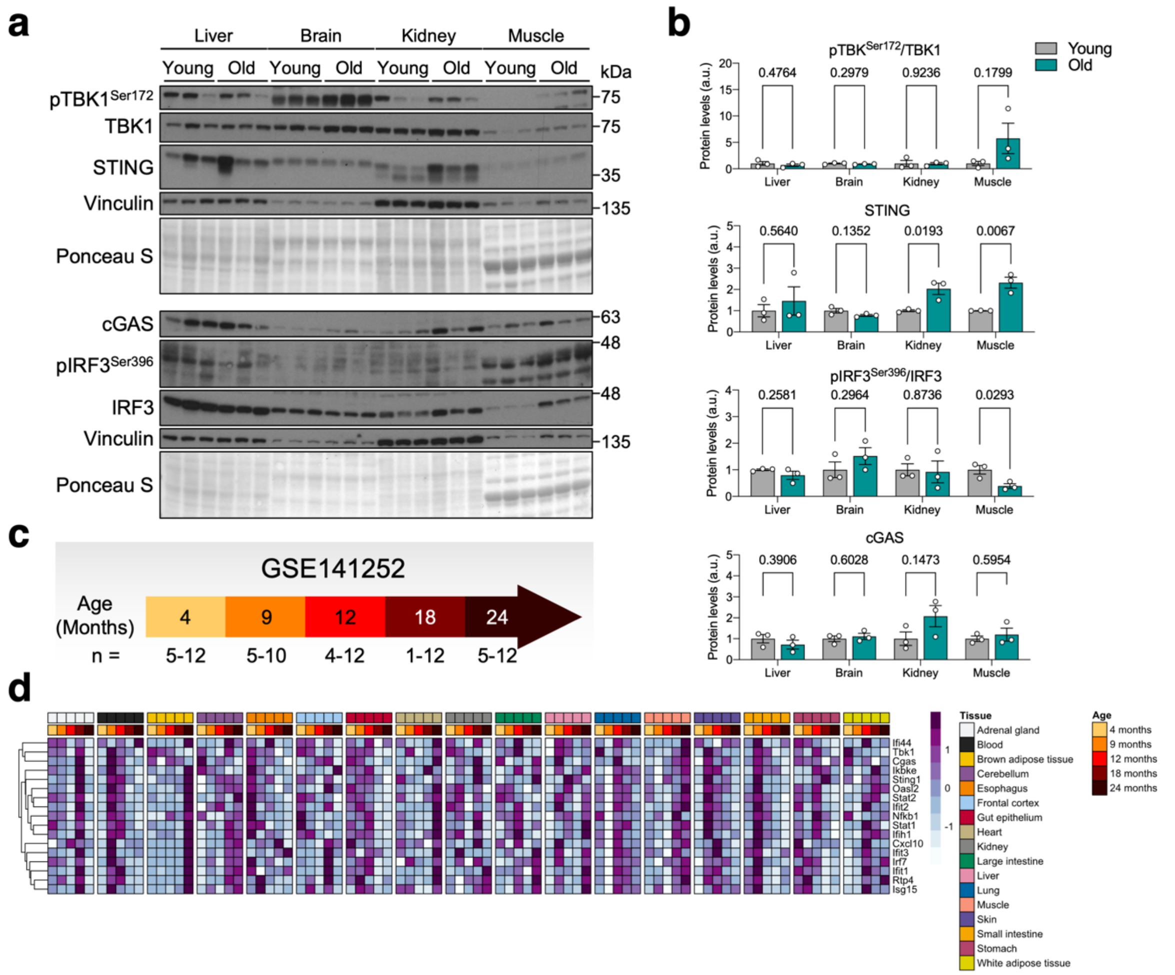

Supplementary Fig. 5. cGAS/STING signaling in other organs during physiological aging. a Western blot analysis of cGAS/STING mediators (TBK1, IRF3, cGAS, STING) in the liver, brain, kidney, and muscle of young and old mice. Protein levels were normalized to loading control (Vinculin in liver, brain, and kidney; Ponceau in muscle). **b** Quantification of protein levels in a (n = 3 mice). **c** Experimental design: bulk RNA-seq of 17 different organs from C57Bl6/J mice collected at 4, 9, 12, 18, or 24 months (GSE141252; Stoeger et al., 2022). **d** Heatmap showing expression of cGAS/STING pathway modulators in samples from **c**. All data are expressed as the mean ± s.e.m. Dots represent individual mice. *P* values were calculated using the two-tailed Student’s *t*-test. Source data are provided as a Source Data file.

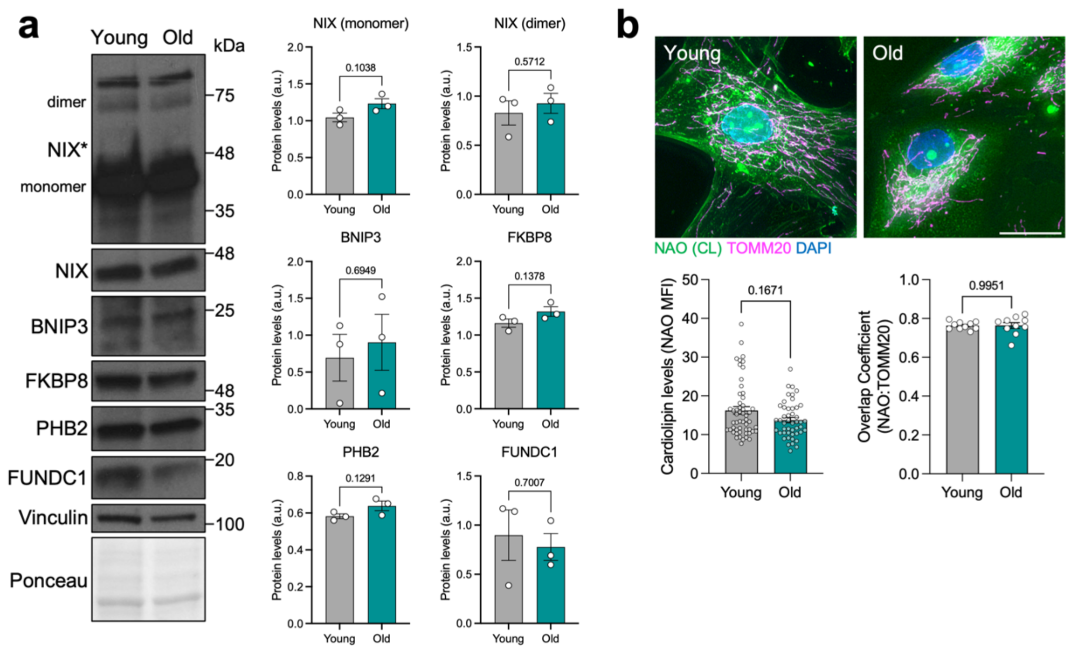

Supplementary Fig. 6. Receptor- and lipid-mediated mitophagy in old NHDFs. a Western blot analysis of mitophagy receptors (BNIP3L/NIX, BNIP3, FKBP8, PHB2, FUNDC1) in NHDFs from young and old donors (*n* = 3 biological replicates). Vinculin was used as a loading control and asterisk indicates higher exposure for chemiluminescence detection. **b** Immunostaining for mitochondria (TOMM20, magenta) and cardiolipin (CL) labelling (NAO, green): representative images and corresponding quantification of CL levels (*n* = 47-54 cells) and TOMM20:CL colocalization (*n* = 10 pictures). Scale bars, 25 µm (**b**). All data are expressed as the mean ± s.e.m. Dots represent individual mice. *P* values were calculated using a two-tailed Mann-Whitney *U*-test (**b**, left) or two-tailed Student's *t*-test (**b**, right). Source data are provided as a Source Data file.

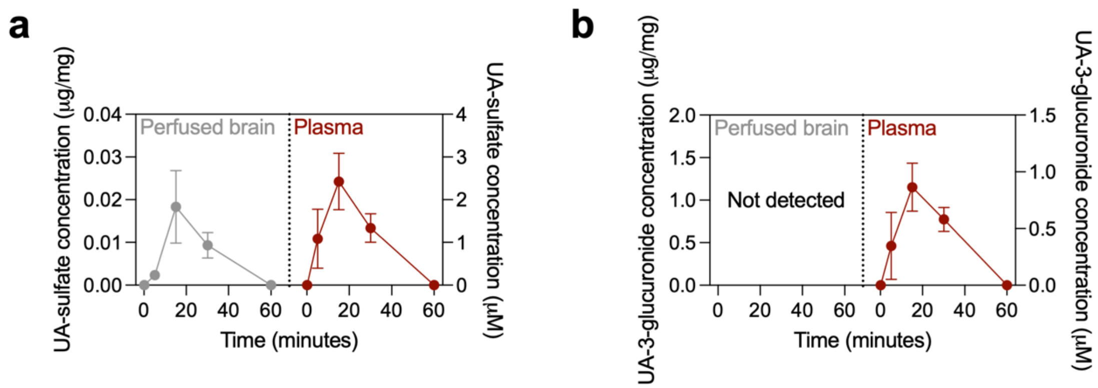

Supplementary Fig. 7. Detection of UA conjugates in perfused brain and plasma samples from C57Bl6/J mice. a Quantification of the levels of conjugated UA-sulfate by UPLC-ESI-QTOF-MS in perfused brain and plasma. **b** Quantification of levels of conjugated UA-3-glucuronide by UPLC-ESI-QTOF-MS in perfused brain (not detected) and plasma. All data are expressed as the mean ± s.e.m. of 2–3 mice per timepoint. Source data are provided as a Source Data file.

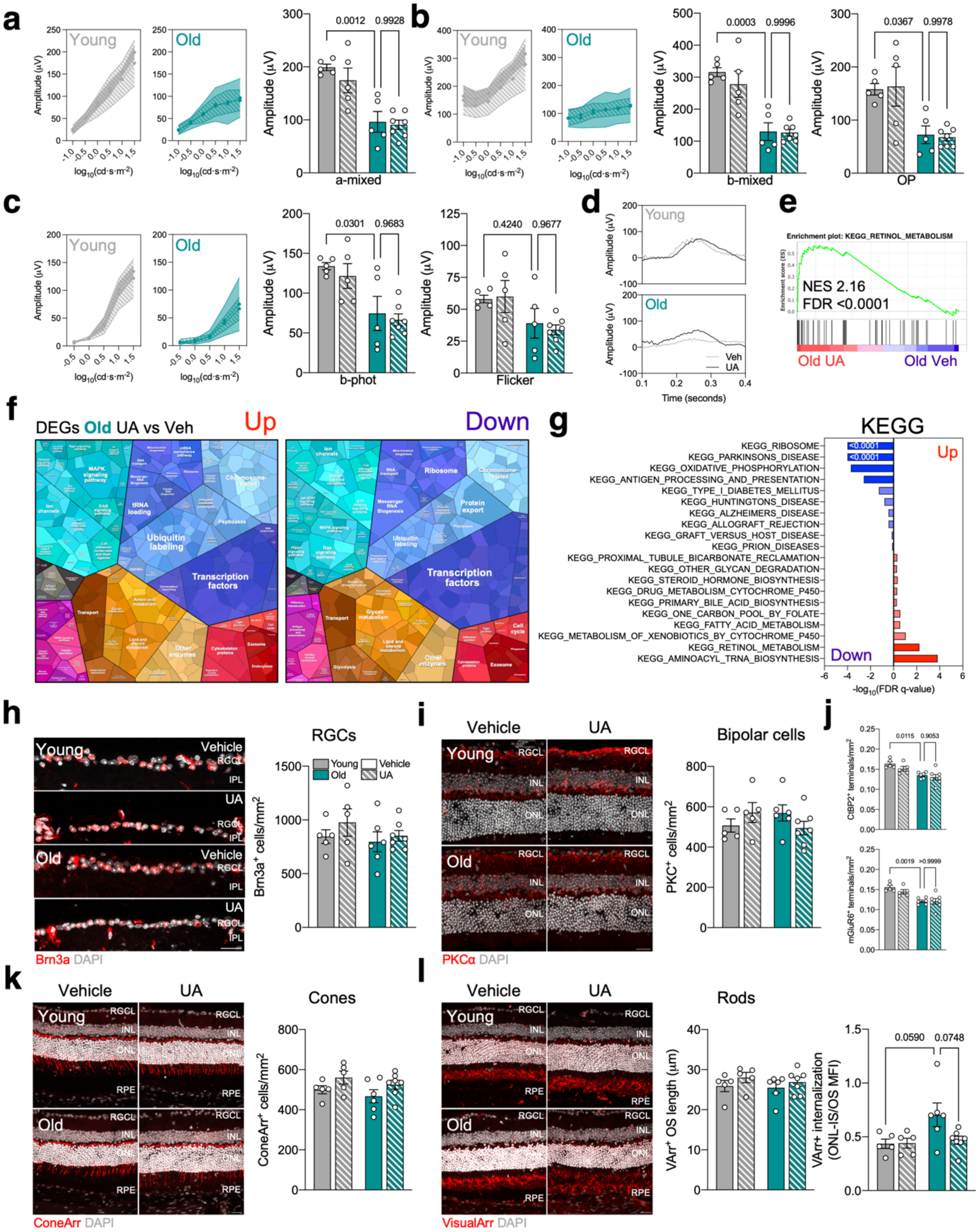

a
Young
Old
Amplitude (µV)
log10(cd·s·m-2)
0.0012
0.9928
a-mixed
b
0.0003
0.9996
b-mixed
0.0367
0.9978
OP
c
0.0301
0.9683
b-phot
0.4240
0.9677
Flicker
d
Veh
UA
Time (seconds)
e
Enrichment plot: KEGG_RETINOL_METABOLISM
NES 2.16
FDR <0.0001
Old UA
Old Veh
f
DEGs Old UA vs Veh
Up
Down
g
KEGG
KEGG_RIBOSOME
KEGG_PARKINSONS_DISEASE
KEGG_OXIDATIVE_PHOSPHORYLATION
KEGG_ANTIGEN_PROCESSING_AND_PRESENTATION
KEGG_TYPE_I_DIABETES_MELLITUS
KEGG_HUNTINGTONS_DISEASE
KEGG_ALZHEIMERS_DISEASE
KEGG_ALLOGRAFT_REJECTION
KEGG_GRAFT_VERSUS_HOST_DISEASE
KEGG_PRION_DISEASES
KEGG_PROXIMAL_TUBULE_BICARBONATE_RECLAMATION
KEGG_OTHER_GLYCAN_DEGRADATION
KEGG_STEROID_HORMONE_BIOSYNTHESIS
KEGG_DRUG_METABOLISM_CYTOCHROME_P450
KEGG_PRIMARY_BILE_ACID_BIOSYNTHESIS
KEGG_ONE_CARBON_POOL_BY_FOLATE
KEGG_FATTY_ACID_METABOLISM
KEGG_METABOLISM_OF_XENOBIOTICS_BY_CYTOCHROME_P450
KEGG_RETINOL_METABOLISM
KEGG_AMINOACYL_TRNA_BIOSYNTHESIS
<0.0001
<0.0001
-log10(FDR q-value)
h
RGCs
Young
Old
Vehicle
UA
Brn3a+ cells/mm2
Brn3a DAPI
i
Bipolar cells
PKC+ cells/mm2
PKCα DAPI
j
0.0115
0.9053
CtBP2+ terminals/mm2
0.0019
>0.9999
mGluR6+ terminals/mm2
k
Cones
ConeArr+ cells/mm2
ConeArr DAPI
l
Rods
VArr+ OS length (µm)
0.0590
0.0748
VArr+ internalization (ONL-IS/OS MFI)
VisualArr DAPI
RGCL
IPL
INL
ONL
RPE

Supplementary Fig. 8. Prolonged UA administration does not induce side effects in the mouse retina. a ERG assessment of visual function (a-wave) in high intensity scotopic conditions and quantification of mixed a-wave amplitude (μV) at 32 cd·s·m^{-2} in young and old mice treated with vehicle or UA (*n* = 5-7 mice). **b** ERG assessment of visual function (b-wave and oscillatory potentials [OP]) in high intensity scotopic conditions and quantification of mixed b-wave and OP amplitude (μV) at 32 cd·s·m^{-2} in young and old mice treated with vehicle or UA (*n* = 5-7 mice). **c** ERG assessment of visual function (b-wave and flicker) in photopic conditions and quantification of mixed b-wave and flicker amplitude (μV) at 32 cd·s·m^{-2} in young and old mice treated with vehicle or UA (*n* = 5-7 mice). **d** Graph depicting scotopic ERG amplitude from **Fig. 4g. e** Enrichment analysis of retinol metabolism (KEGG) using transcriptomic data from old mice treated with vehicle or UA. **f** Geometric Proteomap depicting DEGs that are up- or downregulated in UA-treated old mice compared with vehicle-treated counterparts. Shape size is proportional to the fold-change (minor grid) and number (major grid) of genes associated with the corresponding. **g** Bulk RNA-seq: top enriched KEGG pathways in UA- versus vehicle-treated old mice. **h** Brn3a^{+} immunostaining in retinal ganglion cells (RGCs, red): representative images and corresponding quantification (*n* = 5-7 mice). **i** PKCα^{+} immunostaining of bipolar interneurons (red): representative images and quantification (*n* = 5-7 mice). **j** Quantification of the number of CtBP2^{+} (photoreceptor, pre-synaptic) and mGluR6^{+} (bipolar, post-synaptic) terminals from Fig. 4h (*n* = 5-7 mice). **k** ConeArrestin^{+} immunostaining to identify cone photoreceptors (red): representative images and corresponding quantification (*n* = 5-7 mice). **l** VisualArrestin^{+} immunostaining: representative images and quantification of rod photoreceptor (red) length and internalization (*n* = 5-7 mice). Scale bars, 15 μm (**h**, **j**, **k**, **l**). All data are

expressed as the mean ± s.e.m. Dots represent individual mice. *P* values were calculated using 2-way ANOVA with Tukey's *post-hoc* test. Source data are provided as a Source Data file.

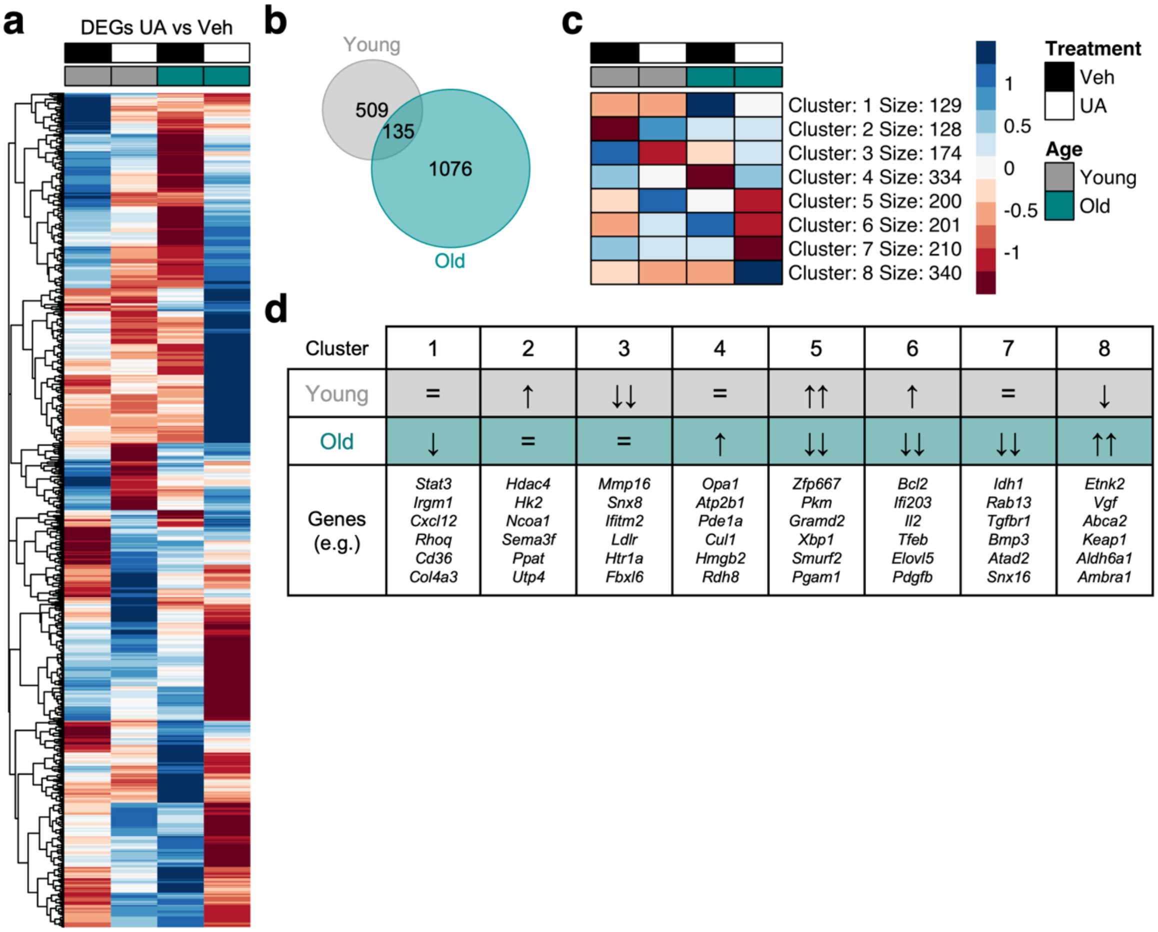

Cluster	1	2	3	4	5	6	7	8
Young	=	↑	↓↓	=	↑↑	↑	=	↓
Old	↓	=	=	↑	↓↓	↓↓	↓↓	↑↑
Genes (e.g.)	*Stat3 Irgm1 Cxcl12 Rhoq Cd36 Col4a3*	*Hdac4 Hk2 Ncoa1 Sema3f Ppat Utp4*	*Mmp16 Snx8 Ifitm2 Ldlr Htr1a Fbxl6*	*Opa1 Atp2b1 Pde1a Cul1 Hmgb2 Rdh8*	*Zfp667 Pkm Gramd2 Xbp1 Smurf2 Pgam1*	*Bcl2 Ifi203 Il2 Tfeb Elovl5 Pdgfb*	*Idh1 Rab13 Tgfbr1 Bmp3 Atad2 Snx16*	*Etnk2 Vgf Abca2 Keap1 Aldh6a1 Ambra1*

Supplementary Fig. 9. Transcriptomic changes with UA treatment are age-dependent. a Heatmap depicting DEGs in young and old mice treated with UA. **b** Venn diagram showing DEGs in the retina of UA-treated young and old mice. **c** *k*-means clustering of DEGs into 8 distinct clusters. **d** Classification of the clusters identified in **c** and examples of corresponding genes.

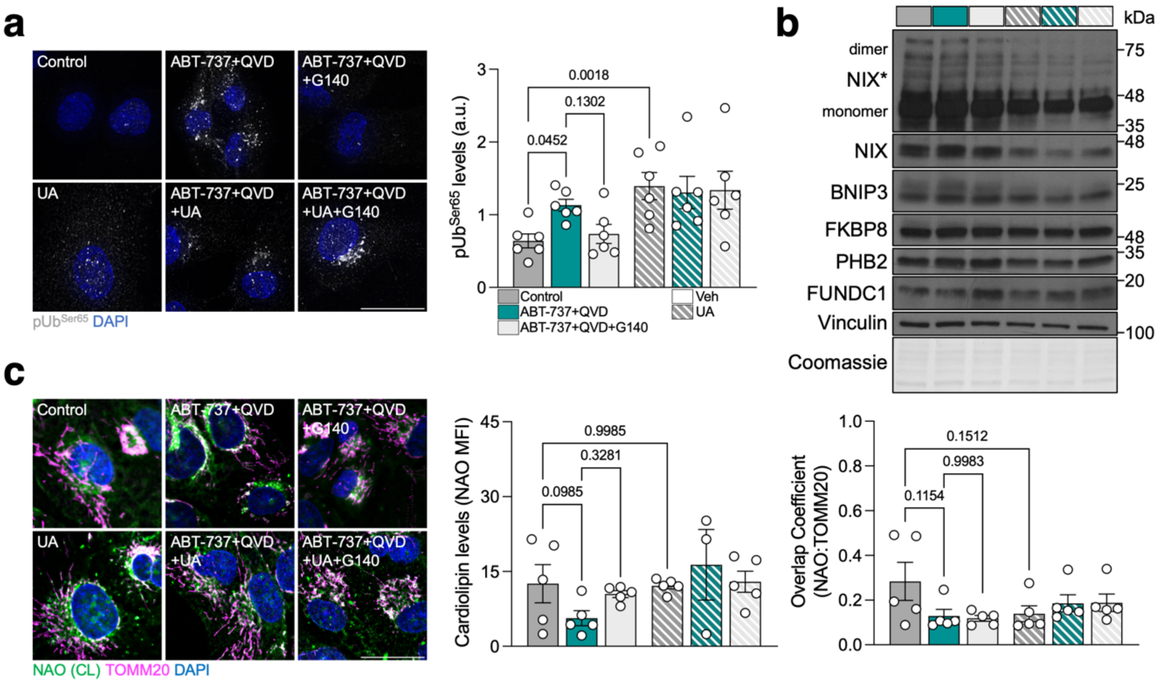

Supplementary Fig. 10. Mitophagy pathways in ARPE-19 cells treated with ABT-737. a Representative images showing phospho-Ubiquitin^{Ser65} immunostaining (gray) in ARPE-19 cells treated with ABT-737 and/or UA, and corresponding quantification (*n* = 5 independent experiments). **b** Western blot analysis of mitophagy receptors (BNIP3L/NIX, BNIP3, FKBP8, PHB2, FUNDC1) in ARPE-19 cells (*n* = representative of 3 independent experiments). Vinculin was used as a loading control and asterisk indicates higher exposure for chemiluminescence detection. **c** Immunostaining for mitochondria (TOMM20, magenta) and cardiolipin (CL) labelling (NAO, green): representative images and corresponding quantification of CL levels and TOMM20:CL colocalization (*n* = 5 independent experiments). Scale bars, 25 µm (**a**, **c**). All data are expressed as the mean ± s.e.m. Dots represent independent experiments (**a**) or images (**c**). *P* values were calculated using a 1-way ANOVA with Šídák's *post-hoc* test (**a**, **c**). Source data are provided as a Source Data file.

Antigen specificity	Species	Dilution	Application	Supplier	Reference
phospho-Ubiquitin(Ser65)	Rabbit	1:200	IF	Merck	ABS1513-I
DNA	Mouse	1:50	IF	Progen	61014
phospho-TBK1(Ser172)	Rabbit	1:1000	WB	Cell Signaling	5483
TBK1	Rabbit	1:1000	WB	Cell Signaling	3504
STING (Mouse)	Rabbit	1:1000	WB	Cell Signaling	50494
cGAS (Mouse)	Rabbit	1:100, 1:1000	IF, WB	Cell Signaling	31659
phospho-IRF3(Ser396)	Rabbit	1:500-1:1000	WB	Cell Signaling	29047
IRF3 (Mouse)	Rabbit	1:1000	WB	Cell Signaling	4302
Vinculin	Rabbit	1:1000	WB	Abcam	ab129002
TOMM20	Rabbit	1:200-1:500	IF	Santa Cruz	sc-11415
CtBP2	Mouse	1:100	IF	BD	612044
mGluR6	Rabbit	1:100	IF	Alomone	AGC-026
4-hydroxynonenal	Rabbit	1:100	IF	Abcam	ab46545
GFAP	Rabbit	1:500	IF	Dako	Z0334
GS	Mouse	1:500	IF	Millipore	MAB302
Iba1	Rabbit	1:100	IF	Wako	019-19741
STING (Human)	Rabbit	1:1000	WB	Cell Signaling	13647
cGAS (Human)	Rabbit	1:500	WB	Cell Signaling	15102
IRF3 (Human)	Rabbit	1:1000	WB	Cell Signaling	11904
BNIP3L/NIX	Rabbit	1:1000	WB	Cell Signaling	12396
BNIP3	Mouse	1:1000	WB	Abcam	ab10433
FKBP8	Rabbit	1:1000	WB	ProteinTech	11173-1-AP
PHB2	Rabbit	1:1000	WB	Thermo Fisher	PA5-14133
FUNDC1	Rabbit	1:1000	WB	Cell Signaling	49240
LC3	Rabbit	1:100	IF	Novus	NB100-2220
LAMP1	Rat	1:100	IF	DSHB	1D4B
SQSTM1/p62	Mouse	1:100	IF	Abcam	ab56416
Ubiquitin (P4D1)	Mouse	1:100	IF	Santa Cruz	sc-8017
NDUFS1	Rabbit	1:1000	WB	Abcam	Ab169540
UQCRC2	Mouse	1:1000	WB	Abcam	Ab14745
MT-CO1	Mouse	1:1000	WB	Invitrogen	459600
SDHB	Mouse	1:1000	WB	Abcam	Ab14714
TIMM23	Mouse	1:1000	WB	BD	611222
ATP5A	Mouse	1:1000	WB	Abcam	ab14748
POU4F1/Brn3a	Mouse	1:100	IF	Millipore	MAB1585
PKCα	Rabbit	1:500	IF	Sigma	P4334
ConeArrestin	Rabbit	1:200	IF	Millipore	AB15282
VisualArrestin	Mouse	1:200	IF	Santa Cruz	sc-166383
Alexa-647 Goat anti-Rabbit	-	1:200	IF	Molecular Probes	A-21244
Alexa-647 Goat anti-Mouse	-	1:200	IF	Molecular Probes	A-21235
Alexa-647 Goat anti-Rat	-	1:200	IF	Molecular Probes	A-21247
Alexa-488 Goat anti-Mouse	-	1:200	IF	Molecular Probes	A-11001
Pacific Blue Goat anti-Rabbit	-	1:200	IF	Invitrogen	P-10994
HRP Goat anti-Rabbit	-	1:2000	WB	Invitrogen	31460
HRP Goat anti-Mouse	-	1:2000	WB	Invitrogen	31430

Supplementary Table 1. Antibodies and dilutions. IF: immunofluorescence; WB: western blot.
