# Supplementary material for: Mitophagy curtails cytosolic mtDNA-dependent activation of cGAS/STING inflammation during aging
Source: Nat Commun. 2024 Jan 27;15:830. doi: 10.1038/s41467-024-45044-1 (PMC10821893; doi:10.1038/s41467-024-45044-1)
Supplement: Supplementary file 3 — Reporting Summary [file 41467_2024_45044_MOESM3_ESM.pdf]

nature portfolio

Corresponding author(s): Patricia Boya

Last updated by author(s): Dec 18, 2023

Reporting Summary

Nature Portfolio wishes to improve the reproducibility of the work that we publish. This form provides structure for consistency and transparency in reporting. For further information on Nature Portfolio policies, see our Editorial Policies and the Editorial Policy Checklist.

Statistics

For all statistical analyses, confirm that the following items are present in the figure legend, table legend, main text, or Methods section.

n/a	Confirmed	
☐	☒	The exact sample size (*n*) for each experimental group/condition, given as a discrete number and unit of measurement
☐	☒	A statement on whether measurements were taken from distinct samples or whether the same sample was measured repeatedly
☐	☒	The statistical test(s) used AND whether they are one- or two-sided<br>*Only common tests should be described solely by name; describe more complex techniques in the Methods section.*
☒	☐	A description of all covariates tested
☐	☒	A description of any assumptions or corrections, such as tests of normality and adjustment for multiple comparisons
☐	☒	A full description of the statistical parameters including central tendency (e.g. means) or other basic estimates (e.g. regression coefficient) AND variation (e.g. standard deviation) or associated estimates of uncertainty (e.g. confidence intervals)
☐	☒	For null hypothesis testing, the test statistic (e.g. *F*, *t*, *r*) with confidence intervals, effect sizes, degrees of freedom and *P* value noted<br>*Give P values as exact values whenever suitable.*
☒	☐	For Bayesian analysis, information on the choice of priors and Markov chain Monte Carlo settings
☒	☐	For hierarchical and complex designs, identification of the appropriate level for tests and full reporting of outcomes
☒	☐	Estimates of effect sizes (e.g. Cohen's *d*, Pearson's *r*), indicating how they were calculated

Our web collection on statistics for biologists contains articles on many of the points above.

Software and code

Policy information about availability of computer code

Data collection

Diagrams were created using BioRender (https://www.biorender.com). Immunofluorescence images were acquired using a Leica SP8 Confocal system or Thunder Imager coupled to Leica LASX software (v3.5.7). qPCR data was collected using LightCycler 480 software (v1.2) Flow cytometry data was collected using a Beckman-Coulter CytoFlex S intrument and CytExpert (v2.3). Oxygen Consumption Rate was analyzed by well respirometry using a Seahorse XFe24 Analyzer and Wave software (v2.6). Electroretinography (ERG) was recorded using LabChart (v8.1.24). Optical coherence tomography (OCT) data was acquired using Heidelberg SPECTRALIS OCT. Electron microscopy images were acquired using a Zeiss EM 902 TEM system. Urolithin metabolite levels were determined using a UPLC-ESI-QTOF-MS system formed by an Agilent 1290 Infinity UPLC coupled to a 6550 Accurate-Mass Quadrupole time-of-flight mass spectrometer and a Poroshell 120 EC-C18 column. ProteoMaps were generated using (https://proteomaps.net).

Data analysis

Statistical analysis was performed using GraphPad Prism (v9.0). Image analysis was perfomed using Fiji (ImageJ) and specific pipelines are described in the Methods section. ERG were analyzed using LabChart Reader (v8.1.22). RNAseq data was processed using R (v4.2.0) by the bioinformatics core unit at IRB Barcelona (Barcelona, Spain). DEGs were analyzed using limma (v3.54.2), enrichment analysis using GSEA (v4.2.3) and heatmaps were generated using pheatmap (v1.0.12).

For manuscripts utilizing custom algorithms or software that are central to the research but not yet described in published literature, software must be made available to editors and reviewers. We strongly encourage code deposition in a community repository (e.g. GitHub). See the Nature Portfolio guidelines for submitting code & software for further information.

Data

Policy information about availability of data

All manuscripts must include a data availability statement. This statement should provide the following information, where applicable:

- Accession codes, unique identifiers, or web links for publicly available datasets
- A description of any restrictions on data availability
- For clinical datasets or third party data, please ensure that the statement adheres to our policy

Datasets for human fibroblasts (GSE113957) and C57BL6/J mice (GSE141252) are publicly available at Gene Expression Omnibus(https://www.ncbi.nlm.nih.gov/gds). Original bulk retina RNAseq from this study has been deposited to GEO with accession number GSE231882.

Research involving human participants, their data, or biological material

Policy information about studies with human participants or human data. See also policy information about sex, gender (identity/presentation), and sexual orientation and race, ethnicity and racism.

Reporting on sex and gender	N/A
Reporting on race, ethnicity, or other socially relevant groupings	N/A
Population characteristics	N/A
Recruitment	N/A
Ethics oversight	N/A

Note that full information on the approval of the study protocol must also be provided in the manuscript.

Field-specific reporting

Please select the one below that is the best fit for your research. If you are not sure, read the appropriate sections before making your selection.

☒ Life sciences ☐ Behavioural & social sciences ☐ Ecological, evolutionary & environmental sciences

For a reference copy of the document with all sections, see nature.com/documents/nr-reporting-summary-flat.pdf

Life sciences study design

All studies must disclose on these points even when the disclosure is negative.

Sample size	Sample size was determined based on preliminary experiments and published data from our group (PMID: 23521856). No power analysis was used to determine sample size.
Data exclusions	For in vivo aging experiments, all animals underwent veterinary examinations before sample collection or intervention study. Mice deemed unhealthy due to an unrelated or pre-existing conditions were excluded from the study.
Replication	Individual data for each mouse is reported, together with the mean and standard error of the mean corresponding to each group. Cell culture experiments included biological and technical replicates, all attempts at replication were sucesful.
Randomization	In the case of the intervention study, mice within each age cohort were randomly assigned to vehicle or drug group. Cell culture experiments with immortalized cell lines or fibroblasts did not involve any randomization other than assigning a given treatment to a specific well; multi-well plate layouts were switched up for different experiments.
Blinding	Behavioural and electrophysiological tests were performed and analyzed by an investigator blind to condition and group. Standardized automatic procedures were used for data collection and analysis of microscopy, flow cytometry, qPCR and respirometry data to prevent bias. Cell type counting, as shown in figure 5 and supplementary figure 8 were performed manually by an investigator blind to condition and treatment group.

Reporting for specific materials, systems and methods

We require information from authors about some types of materials, experimental systems and methods used in many studies. Here, indicate whether each material, system or method listed is relevant to your study. If you are not sure if a list item applies to your research, read the appropriate section before selecting a response.

Materials & experimental systems

n/a	Involved in the study
☐	☒ Antibodies
☐	☒ Eukaryotic cell lines
☒	☐ Palaeontology and archaeology
☐	☒ Animals and other organisms
☒	☐ Clinical data
☒	☐ Dual use research of concern
☒	☐ Plants

Methods

n/a	Involved in the study
☒	☐ ChIP-seq
☐	☒ Flow cytometry
☒	☐ MRI-based neuroimaging

Antibodies

Antibodies used

phospho-Ubiquitin(Ser65) Rabbit 1:200 IF Merck ABS1513-I
DNA Mouse 1:50 IF Progen 61014
phospho-TBK1(Ser172) Rabbit 1:1000 WB Cell Signaling 5483
TBK1 Rabbit 1:1000 WB Cell Signaling 3504
STING (Mouse) Rabbit 1:1000 WB Cell Signaling 50494
cGAS (Mouse) Rabbit 1:100, 1:1000 IF, WB Cell Signaling 31659
phospho-IRF3(Ser396) Rabbit 1:500-1:1000 WB Cell Signaling 29047
IRF3 (Mouse) Rabbit 1:1000 WB Cell Signaling 4302
Vinculin Rabbit 1:1000 WB Abcam ab129002
TOMM20 Rabbit 1:200-1:500 IF Santa Cruz sc-11415
CtBP2 Mouse 1:100 IF BD 612044
mGluR6 Rabbit 1:100 IF Alomone AGC-026
4-hydroxynonenal Rabbit 1:100 IF Abcam ab46545
GFAP Rabbit 1:500 IF Dako Z0334
GS Mouse 1:500 IF Millipore MAB302
Iba1 Rabbit 1:100 IF Wako 019-19741
STING (Human) Rabbit 1:1000 WB Cell Signaling 13647
cGAS (Human) Rabbit 1:500 WB Cell Signaling 15102
IRF3 (Human) Rabbit 1:1000 WB Cell Signaling 11904
BNIP3L/NIX Rabbit 1:1000 WB Cell Signaling 12396
BNIP3 Mouse 1:1000 WB Abcam ab10433
FKBP8 Rabbit 1:1000 WB ProteinTech 11173-1-AP
PHB2 Rabbit 1:1000 WB Thermo Fisher PA5-14133
FUNDC1 Rabbit 1:1000 WB Cell Signaling 49240
LC3 Rabbit 1:100 IF Novus NB100-2220
LAMP1 Rat 1:100 IF DSHB 1D4B
SQSTM1/p62 Mouse 1:100 IF Abcam ab56416
Ubiquitin (P4D1) Mouse 1:100 IF Santa Cruz sc-8017
NDUFS1 Rabbit 1:1000 WB Abcam Ab169540
UQCRC2 Mouse 1:1000 WB Abcam Ab14745
MT-CO1 Mouse 1:1000 WB Invitrogen 459600
SDHB Mouse 1:1000 WB Abcam Ab14714
TIMM23 Mouse 1:1000 WB BD 611222
ATP5A Mouse 1:1000 WB Abcam ab14748
POU4F1/Brn3a Mouse 1:100 IF Millipore MAB1585
PKCα Rabbit 1:500 IF Sigma P4334
ConeArrestin Rabbit 1:200 IF Millipore AB15282
VisualArrestin Mouse 1:200 IF Santa Cruz sc-166383
Alexa-647 Goat anti-Rabbit - 1:200 IF Molecular Probes A-21244
Alexa-647 Goat anti-Mouse - 1:200 IF Molecular Probes A-21235
Alexa-647 Goat anti-Rat - 1:200 IF Molecular Probes A-21247
Alexa-488 Goat anti-Mouse - 1:200 IF Molecular Probes A-11001
Pacific Blue Goat anti-Rabbit - 1:200 IF Invitrogen P-10994
HRP Goat anti-Rabbit - 1:2000 WB Invitrogen 31460
HRP Goat anti-Mouse - 1:2000 WB Invitrogen 31430

Validation

All antibodies were validated by the manufacturer and other users (https://www.citeab.com). For immunofluorescence experiments negative controls (only 2ry antibody) were included.

Eukaryotic cell lines

Policy information about cell lines and Sex and Gender in Research

Cell line source(s)

Normal human dermal fibroblasts (NHDFs), isolated from adult abdominal dermal explants from 28-year-old (Young, DFM050410A) and 68-year-old (Old, DFM050510B) female donors, were purchased from ZenBio (ZenBio, USA). ARPE-19 cells were obtained from ATCC (CRL-2302) and spontaneously immortalized from a male donor.

Authentication	Fibroblast senescence was assessed using ß-galactosidase activity. ARPE-19 were not validated but presented morphology and features resembling those shown in the literature.
Mycoplasma contamination	Both primary cell lines had been previously tested negative for HIV-1, HIV-2, HTLV-1, HTLV2, hepatitis B, hepatitis C, and mycoplasma. ARPE-19 cells were periodically tested for mycoplasma contamination using imunofluorescence and PCR, all tests came back negative.
Commonly misidentified lines (See ICLAC register)	No commonly misidentified cell lines were used in this study.

Animals and other research organisms

Policy information about studies involving animals; ARRIVE guidelines recommended for reporting animal research, and Sex and Gender in Research

Laboratory animals	C57BL6/J mice wild-type or mito-QC were used for all experiments. All experiments were performed using young (6-8 months) and old (22-26 months) mice in parallel, male and female mice were included in all experiments. Mice were bred and housed at the CIB animal facility, on a 12/12 h light/dark cycle with ad libitum access to food (standard chow) and water. Animals were monitored daily by animal facility technicians and underwent regular veterinary exams.
Wild animals	No wild animals were used in this study
Reporting on sex	Male and female mice were included in all experiments, no variability was observed between sexes and the findings apply to both of them.
Field-collected samples	No field-collected samples were used in this study.
Ethics oversight	All animal experiments were performed according to RU guidelines and the ARVO Statement for the Use of Animals in Ophthalmic and Vision Research, and were authorized by the Centro de Investigaciones Biologicas Margarita Salas (CSIC) institutional bioethics committee and Comunidad de Madrid (PROEX 154.3/21).

Note that full information on the approval of the study protocol must also be provided in the manuscript.

Plants

Seed stocks	*Report on the source of all seed stocks or other plant material used. If applicable, state the seed stock centre and catalogue number. If plant specimens were collected from the field, describe the collection location, date and sampling procedures.*
Novel plant genotypes	*Describe the methods by which all novel plant genotypes were produced. This includes those generated by transgenic approaches, gene editing, chemical/radiation-based mutagenesis and hybridization. For transgenic lines, describe the transformation method, the number of independent lines analyzed and the generation upon which experiments were performed. For gene-edited lines, describe the editor used, the endogenous sequence targeted for editing, the targeting guide RNA sequence (if applicable) and how the editor was applied.*
Authentication	*Describe any authentication procedures for each seed stock used or novel genotype generated. Describe any experiments used to assess the effect of a mutation and, where applicable, how potential secondary effects (e.g. second site T-DNA insertions, mosiacism, off-target gene editing) were examined.*

Flow Cytometry

Plots

Confirm that:

☒ The axis labels state the marker and fluorochrome used (e.g. CD4-FITC).

☒ The axis scales are clearly visible. Include numbers along axes only for bottom left plot of group (a 'group' is an analysis of identical markers).

☒ All plots are contour plots with outliers or pseudocolor plots.

☒ A numerical value for number of cells or percentage (with statistics) is provided.

Methodology

Sample preparation	No immunophenotyping was performed, only intraccelular indicator probes were analyzed.Cells were washed with PBS, trypsinized for 2-3 min and collected by centrifugation at 1200 x g for 5 min. Cells were resuspended in complete medium containing 100nM MitoTracker Green (M7514, Invitrogen) and 5µM MitoSOX Red (M36008, Invitrogen) and incubated for 15 minutes at 37ºC. DAPI (1µg/µL) was added for dead cell exclusion and tubes were kept in ice until analysis.
Instrument	CytoFlex S (V4-B2-Y4-R3; Beckman Coulter).
Software	CytExpert (v2.3).

Cell population abundance	At least 10000 events per sample were collected.
Gating strategy	Cells were identified using FSC/SSC and doublet discrimination was performed using FSC-A/FSC-W. Viable population is identified as DAPI-negative and MitoTracker Green and mitoSOX Red mean fluorescence in the viable population are reported. No immunophenotyping or complex gating strategy was used.

☐ Tick this box to confirm that a figure exemplifying the gating strategy is provided in the Supplementary Information.
